# Supplementary material for: Can neoadjuvant chemoradiotherapy be omitted in cT2N+ and cT3 mid-rectal cancer: Protocol for a prospective, observational, cohort study (CANO)
Source: PLoS One. 2025 Nov 5;20(11):e0321819. doi: 10.1371/journal.pone.0321819 (PMC12588449; doi:10.1371/journal.pone.0321819)
Supplement: Attachment — Submitted filename: Response to reviewers.docx [file pone.0321819.s004.docx]

**Responses to the Comments**

**Reviewer #1:**

Dear reviewer,
Thank you for your substantial contributions. We have attempted to revise our protocol accordingly. Please find detailed explanations below. We hope that we have been able to address the limitations of our study.

Kind regards

1- The aim of minimizing toxicity in rectal cancer treatment by avoiding unnecessary radiotherapy or chemotherapy is a good one. Unfortunately, your non-randomised approach does not seem accurate enough to provide robust data. The non-randomized decisions made by the 'multidisciplinary council' will lead to unadjustable bias.

**Response:** We fully acknowledge this limitation and have strengthened our statistical analysis plan accordingly. Statistical methods have been revised in the manuscript and highlighted in yellow.
To address potential selection bias, we will estimate propensity using logistic regression with relevant covariates and adjust for the propensity score in the primary Cox regression analysis. Additionally, we will conduct sensitivity analyses using both propensity score matching (1:1 nearest neighbor) and inverse probability of treatment weighting (IPTW) to further mitigate bias and ensure the robustness of our results. Although this cannot completely eliminate bias, we believe that these methods can minimize this limitation in an observational study aiming to reflect real-world data.

2- Furthermore, your inclusion criteria did not recognize risk parameters such as EMVI or lateral lymph nodes.

**Response:** We assumed that, in the presence of these factors, the MDT would routinely decide on neoadjuvant therapy. However, you are right, and we have now included these factors in the inclusion and exclusion criteria.

3- You do not perform MSS/MSI testing before treatment what should be mandatory.

**Response:** Unfortunately, MSI testing is not yet performed as a standard in endoscopic pathology in Türkiye. Many centers offer it as an additional test, which is time consuming and usually not covered by insurance and results in extra costs for patients. Therefore, MSI is only tested in endoscopic pathology if the center routinely tests it or if the patient consents to be tested in another institution with extra costs. For this reason, we could not specify unknown pre-treatment MSI status as an exclusion criterion.

However, MSI testing is routinely performed in final surgical pathology in our country. Patients found to have MSI (+) in surgical pathology will not be included in the final analysis. Similarly, patients who had preoperative MSI testing (if performed), were found positive, and/or received immunotherapy will also be excluded from the final analysis. We have revised our flow chart and protocol accordingly to reflect this approach. Also, we have discussed these issues in limitations section.

4- As surgery seems to be mandatory in your trial, the trend towards organ preservation is not acknowledged in your trial.

5- You did not describe whether restaging will be performed after NCRT. In addition, the follow-up scheme, even if it is considered standard, should be described in your paper.

**Response:** Our database includes restaging and follow-up data and the dates of the examinations detailly. These issues have been added to the methods section in separate paragraphs in the revised manuscript. Thank you.

**Reviewer #2:** 

Dear reviewer,

Thank you for your valuable comments. We have tried to address all in the revised version

Kind regards

1. MRI Evaluation Consistency:
Given the central role of MRI in risk stratification, the protocol should address how inter-institutional variability in MRI interpretation will be minimized. Consider including provisions such as standardized educational sessions or the use of reference image sets to ensure consistency across sites.

**Response:** In our study, we aim to observe real-world outcomes. Due to logistical challenges, we are unable to perform centralized radiological and pathological assessments. However, our study website includes a frequently asked questions (FAQ) section where the standards for pathology and MRI reports are clearly described (https://arastirma.tkrcd.org.tr/cano).

Furthermore, during the national Zoom meeting held on June 23, 2025, with centers interested in participating, the radiological and pathological standards were shared with all potential participants. It was also emphasized during the meeting—and published on the website—that centers unable to meet these standards would be offered independent radiologic/pathologic evaluation and MDT (support by TSCRS. We are including this statement in our protocol as well. Thank you.

2. Statistical Methods:
Further details are warranted regarding the planned multivariable regression analysis. Specifically, please clarify:
• The covariates to be included in the model
• Whether a Cox proportional hazards model will be used
• How the two treatment strategies will be compared—e.g., via propensity score adjustment, matching, or inverse probability weighting

**Response:** Thank you very much for your valuable and constructive comments. We have rewritten the statistical analysis section in the manuscript. Briefly;

Age, sex, clinical T and N stage, tumor differentiation, neoadjuvant treatment regimen, interval between RT and surgery, comorbidities, performance status, postoperative complications, tumor histology, pathologic TME quality, and center effect. Cox proportional hazards models will be used for time-to-event outcomes (DFS, OS). Direct TME vs. nCRT+TME will be compared using propensity score adjustment in the primary analysis, with sensitivity analyses using propensity score matching and IPTW.

3. Heterogeneity in Neoadjuvant Treatment (nCRT vs TNT):
The inclusion of both conventional neoadjuvant chemoradiotherapy (nCRT) and total neoadjuvant therapy (TNT) could introduce heterogeneity in the analysis. As TNT has been associated with improved DFS in some studies, how do the authors plan to account for or stratify the impact of TNT separately from standard nCRT?

**Response:** In the final analysis, we will also perform subgroup analyses comparing different neoadjuvant treatment regimens with each other.
